# Discussion on the causes of thrombolysis failure in a patient with STEMI: a case report

**DOI:** 10.1186/s12872-022-02922-0

**Published:** 2022-11-08

**Authors:** Lingzhi Qiu, Jia Li, Hua Yan, Hui Guo, Dan Song, Xi Su

**Affiliations:** 1 Medical College of Wuhan University of Science and Technology, Wuhan, China; 2 Department of Cardiology, Wuhan Asia Heart Hospital, Wuhan, China

**Keywords:** Spontaneous coronary artery dissection, ST-segment elevation myocardial infarction, Thrombolytic therapy, Coronavirus disease 2019, Case report

## Abstract

**Background:**

Spontaneous coronary artery dissection (SCAD) has emerged as an increasingly diagnosed cause of ST-segment elevation myocardial infarction (STEMI), which is easily missed or delayed. The effective use of coronary angiography (CAG) and advanced intracoronary imaging examinations in STEMI patients has led to increased detection of SCAD.

**Case presentation:**

A 59-year-old woman with acute angina pectoris was diagnosed with STEMI detected by electrocardiography combined with measurement of myocardial enzymes. Due to the ongoing pandemic of coronavirus disease 2019 (COVID-19) in Wuhan, she was first given thrombolytic therapy after excluding contraindications according to the requirements of the current consensus statement; however, subsequently, both the symptoms of ongoing chest pain and the electrocardiographic results indicated the failure of thrombolytic therapy, so the intervention team administered rescue percutaneous coronary intervention treatment under third-grade protection. CAG confirmed total occlusion in the distal left anterior descending (LAD) artery, with thrombolysis in myocardial infarction (TIMI) 0 flow, whereas the left circumflex and right coronary arteries appeared normal, with TIMI 3 flow. Intravenous ultrasound (IVUS) was further performed to investigate the causes of occlusion, which verified the absence of atherosclerosis but detected SCAD with intramural haematoma. During the operation, the guidewire reached the distal end of the LAD artery smoothly, the balloon was dilated slightly, and the reflow of TIMI blood could be seen by repeated CAG. During the follow-up period of one and a half years, the patient complained of occasional, slight chest tightness. The repeated CAG showed that the spontaneous dissection in the LAD artery had healed well, with TIMI 3 flow. The repeated IVUS confirmed that the SCAD and intramural haematoma had been mostly resorbed and repaired.

**Conclusion:**

This was a case of failed STEMI thrombolysis in our hospital during the outbreak of COVID-19. This case indicates that doctors need to consider the cause of the disease when treating STEMI patients, especially patients without traditional cardiovascular risk factors. Moreover, CAG and intracoronary imaging examinations should be actively performed to identify the aetiology and improve the treatment success rate.

## Background

Spontaneous coronary artery dissection (SCAD) is a relatively uncommon but not insignificant cause of acute coronary syndrome (ACS), which arises from the spontaneous separation of an epicardial coronary artery wall [1]. The concomitant formation of an intramural haematoma leads to compression of the true lumen, resulting in myocardial ischaemia [2]. SCAD can manifest as angina pectoris, ACS, or even sudden cardiac death. It is now estimated that SCAD is the underlying cause of 1.7 to 4% of ACS and accounts for 0.5% of sudden cardiac deaths [3]. SCAD predominantly afflicts young to middle-aged people with few traditional risk factors for ACS. Although the aetiology remains unclear, SCAD has been demonstrated to be associated with pregnancy, sex hormone levels, fibromuscular dysplasia (FMD), strong mood changes, depression, and so on [4].

At the end of 2019, coronavirus disease 2019 (COVID-19) broke out in Wuhan, which substantially increased the difficulty of treating patients with cardiovascular diseases [5]. With a robust capacity for human-to-human transmission, quick disease progression in some cases, and no specific drug treatment, COVID-19 has placed a certain degree of pressure on the psychological health of the public. To limit nosocomial cross-infection during the epidemic, the guidelines [6] recommended that thrombolytic therapy should be the first-line therapy for patients with ST-segment elevation myocardial infarction (STEMI). Especially for patients with coronary artery obstruction caused by atherosclerotic plaque rupture or intraluminal thrombus, thrombolytic therapy has a better effect. However, thrombolytic therapy is contraindicated in SCAD, as it may propagate dissection and lead to coronary rupture and cardiac tamponade [7].

This paper reports the case of a STEMI patient who was initially considered to have atherosclerotic ACS, but both her ongoing symptoms and the repeated electrocardiogram (ECG) revealed the failure of thrombolytic therapy. Coronary angiography (CAG) revealed total occlusion in the distal left anterior descending (LAD) segment with thrombolysis in myocardial infarction (TIMI) 0 flow. Finally, the case was confirmed as SCAD with intramural haematoma by intravenous ultrasound (IVUS) examination, followed by conservative treatment with drugs. By analysing this case, we emphasize that thrombolytic therapy is not recommended without the exclusion of SCAD, so as not to delay or aggravate the disease for this kind of female patient, especially when they have recently had excessive mental stress. We should make full use of CAG and intracoronary imaging examinations to determine the cause of STEMI and formulate appropriate treatment strategies for this kind of patient.

## Case presentation

A 59-year-old female patient was admitted to our hospital due to acute chest pain for more than 2 hours. At approximately 23:55 on February 28, 2020, she felt severe central chest pain with associated diaphoresis and radiation to the back. She seemed to have no traditional cardiovascular risk factors, whereas she had a prior history of anxiety and panic attacks.

**Fig. 1:**
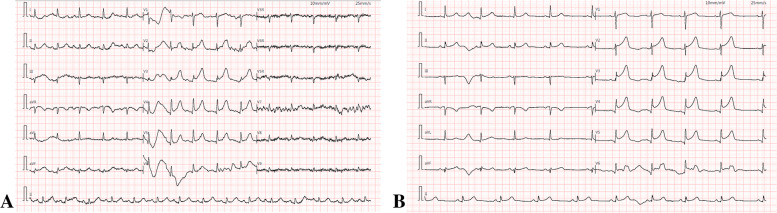
ECG at admission (**A**) and after thrombolysis (**B**)

Her physical examination was unremarkable, and her ECG showed ST-segment elevation of 0.05–0.15 mV in leads V_2–6_ (Fig. 1 A). Her point-of-care ultrasound showed that the motion amplitude of the left ventricular apical wall was decreased, the basal segment of the ventricular septum was thickened, and the left ventricular systolic function was slightly decreased with a left ventricular ejection fraction (LVEF) of 46%. Cardiac troponins were elevated [serum troponin I: 6.168 ng/ml (normal range < 0.03 ng/ml)].

A diagnosis of STEMI was made in the ambulance, followed by oral administration of aspirin (300 mg), ticagrelor (180 mg), atorvastatin (40 mg), and metoprolol (5 mg), and nitroglycerine (10 μg/min) was given intravenously immediately in this emergency. Heparin anticoagulation therapy was added (the load was 50 U/kg, and then 12 U/min was pumped intravenously to maintain APTT from 50 s to 70 s). The patient was transferred to the coronary care unit after the exclusion of COVID-19 by lung CT imaging and IgM testing and, later, by COVID-19 nucleic acid testing. Because of her severe chest pain after admission, morphine was administered for sedation, and acid-suppressive medication was given to inhibit acid and protect the stomach. After excluding the complications according to the requirements of the current consensus statement, alteplase thrombolysis was administered at 03:30 (rT-PA 50 mg dissolved in 50 ml NS; 8 ml was statically pushed and pumped at a speed of 28 ml/h) and ended at 04:30. Thrombolysis was considered unsuccessful because the degree of chest pain was not alleviated and the ST segment of ECG did not decrease (Fig. 1B).

**Fig. 2:**
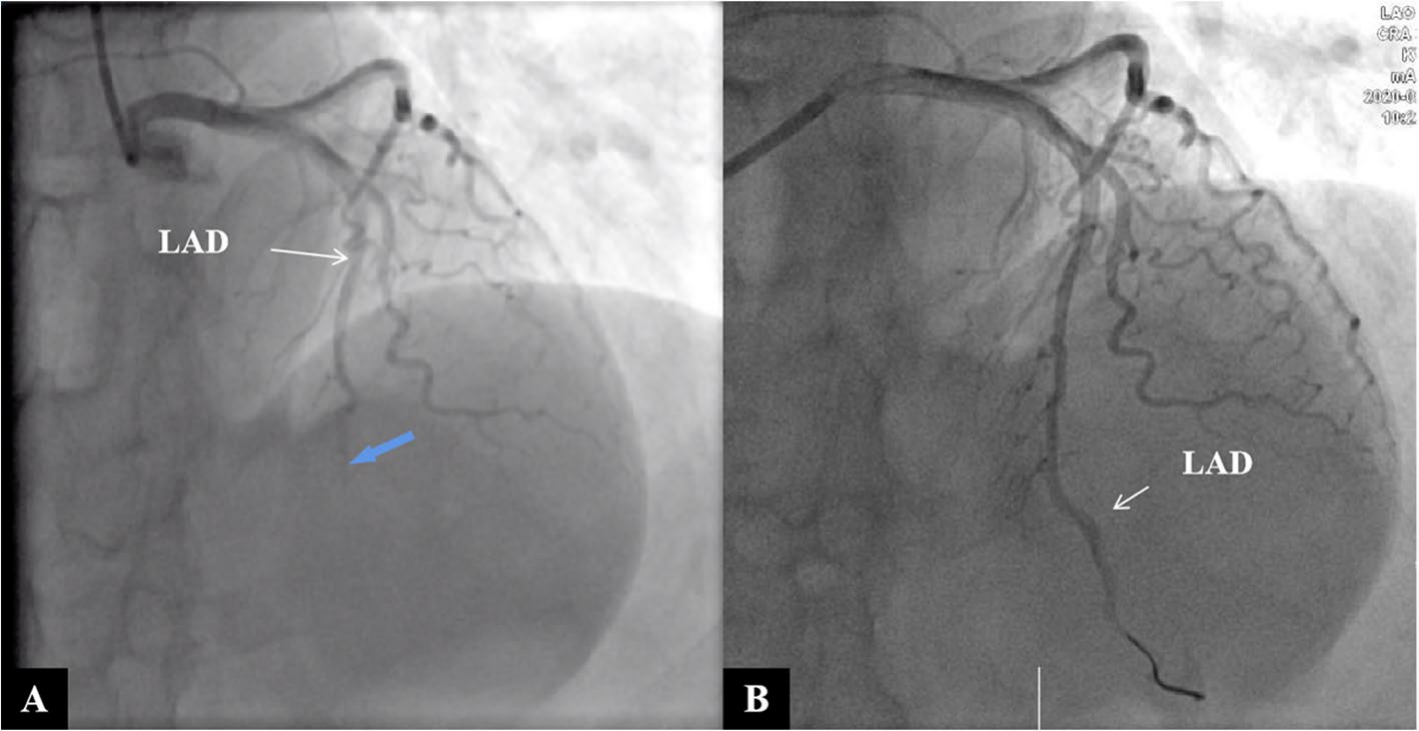
CAG demonstrated total occlusion in the distal LAD segment (blue arrow in **A**), with TIMI 0 flow. The guidewire reached the distal end of the LAD lesion smoothly, and repeated CAG showed that blood was reflowing through the distal LAD segment (TIMI 3 flow) (**B**)

**Fig. 3:**
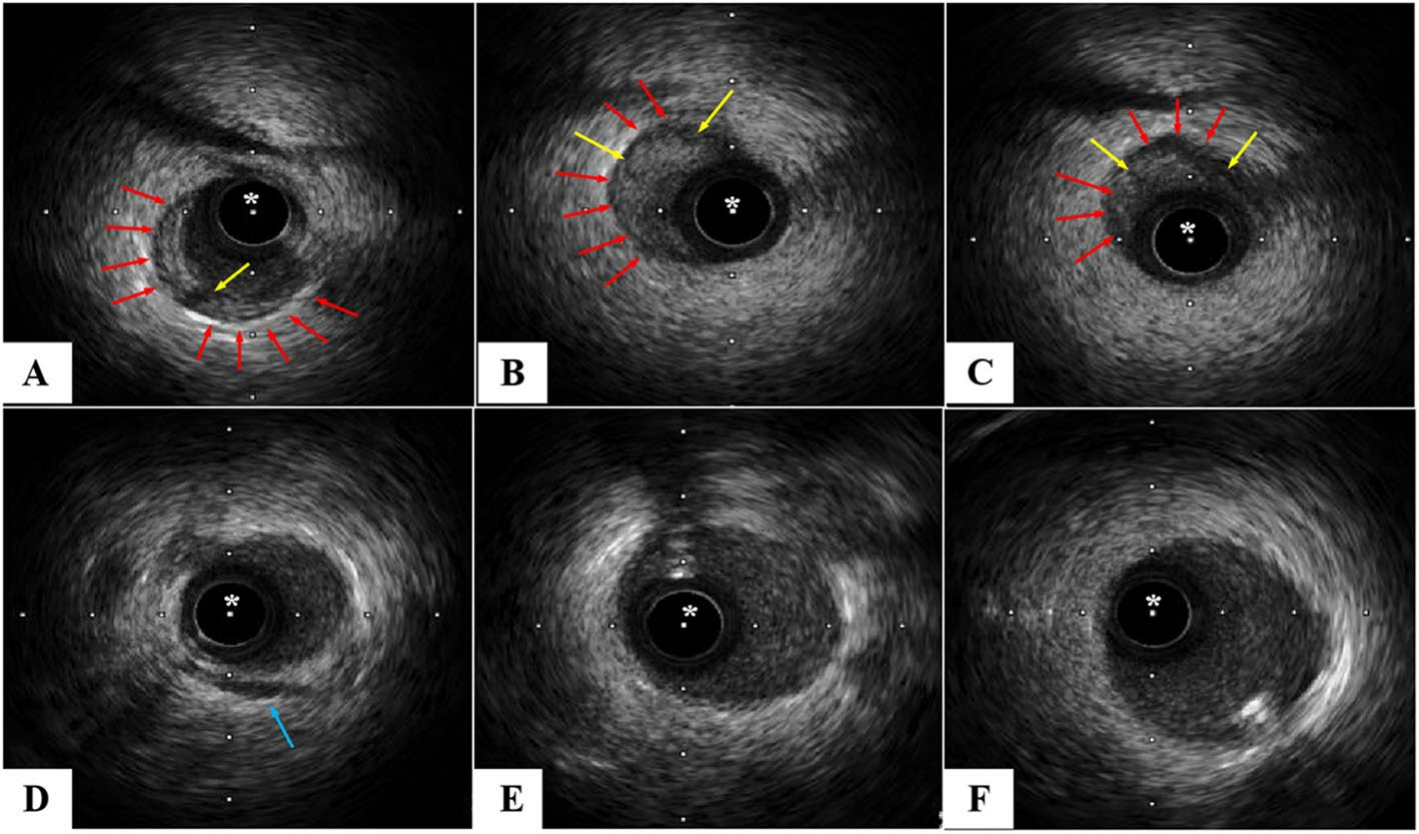
The first three images show the narrowing of the lumen caused by haematoma compression, whereas the last three images show normal blood vessels. The IVUS catheter (asterisk) was withdrawn from the distal end of the LAD, and the IVUS catheter was located in the true lumen of the blood vessel. Spiral dissection with haematoma could be seen in the distal segment of LAD, while the proximal and middle vessels were normal. No atherosclerotic plaque was seen. The red arrow in the picture shows spiral dissection with haematoma, involving 5:00 to 10:00, 6:00 to 12:00, and 8:00 to 3:00, respectively, and the yellow arrow shows spiral dissection. The blue arrow shows the myocardial bridge

Next, our intervention team remedied CAG under third-grade protection, which showed total occlusion in the distal segments of the LAD, with TIMI 0 flow, whereas the left circumflex and right coronary arteries appeared normal, with TIMI 3 flow (Fig. 2A). During the operation, the guidewire reached the distal end of the LAD lesion smoothly, the balloon dilated slightly, and the reflow of TIMI blood could be seen by repeated CAG (Fig. 2B). The operator considered that there were no plaques in other blood vessels and that the patient had no conventional risk factors for ACS, such as hypertension, diabetes, hyperlipemia, smoking, etc. IVUS was performed to explore the aetiology and guide the treatment, which showed SCAD with an intramural haematoma at the distal segment of the LAD branch. The compression of the haematoma resulted in lumen occlusion (Fig. 3). Therefore, SCAD was diagnosed. No stent implantation was performed, and the patient commenced dual antiplatelet therapy (aspirin and ticagrelor) followed by long-term antianginal pharmacologic therapy (nitrates, calcium-channel blockers, and beta-blockers). She remained clinically stable and was subsequently discharged from the hospital on March 09, 2020.

**Fig. 4:**
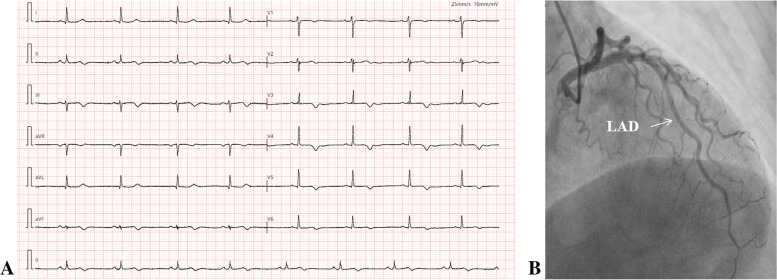
Repeated ECG (**A**) revealed the return of the ST segment to the baseline level, the r wave in the precordial area was poorly increased, and the T wave was inverted or bidirectional. Repeated CAG (**B**) showed no occlusion in the distal LAD segment, with TIMI 3 flow

**Fig. 5:**
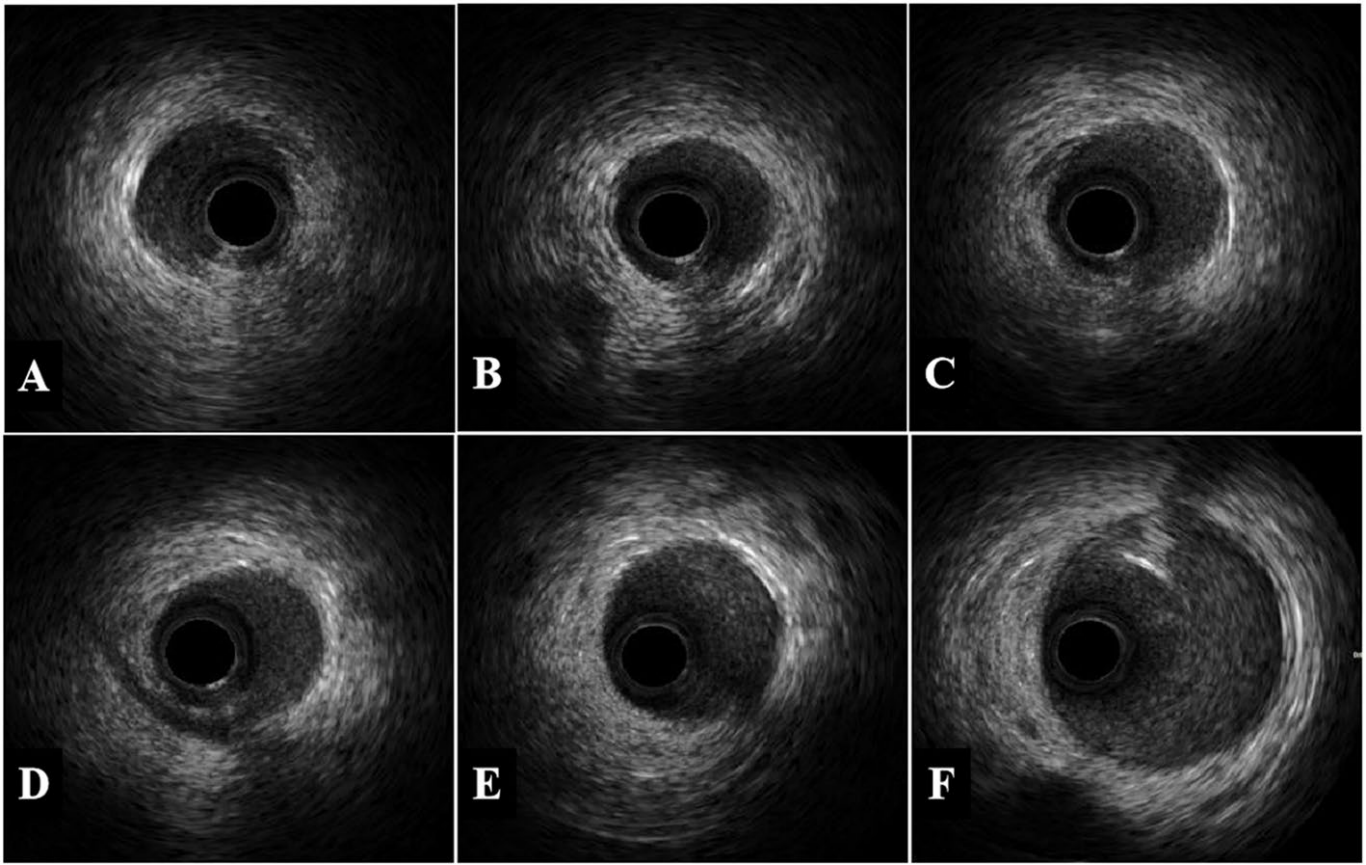
The IVUS catheter was withdrawn from the distal end of the LAD (due to the slender distal end, IVUS failed to evaluate the near apical segment of the LAD), and the results showed that the spontaneous dissection in the distal LAD segment had healed well and that the haematoma had been resorbed. Residual haematoma may have remained in the distal area near the apical segment of the LAD, with mild stenosis

On August 26, 2021, the patient was readmitted to the hospital after one and a half years. She complained of occasional chest tightness and discomfort, and there were no positive signs on cardiopulmonary physical examination. The ECG revealed the return of the ST segment to the baseline level, the R wave in the precordial area was poorly increased, and the T wave was inverted or bidirectional (Fig. 4A). The repeated CAG showed that the dissection in the distal segments of the LAD had healed well, with TIMI 3 flow (Fig. 4B). The repeated IVUS confirmed that the dissection and intramural haematoma had been mostly resorbed and repaired. There was no obvious atherosclerotic plaque, and the distal end of the blood vessel was slender (Fig. 5).

## Discussion

SCAD has currently gained attention as a nonnegligible cause of STEMI, which refers to a nontraumatic, noniatrogenic separation of the wall of epicardial coronary arteries [8]. Because the recognition of SCAD is still very limited, there exists a tendency towards underdiagnosis or even misdiagnosis. SCAD affects a different patient population than atherosclerotic disease does, and the pathophysiology and management are also different [9].

The precipitating factors of patients with SCAD remain unclear. SCAD is likely influenced by a combination of factors that include sex; hormonal fluctuations; underlying arteriopathies; genetics; and environmental, physical, and emotional precipitants [10]. SCAD is associated with high levels of psychological distress, including depression, anxiety, and posttraumatic stress disorder, and after STEMI, women bear a greater burden of these symptoms. Emotional stressors appear to be more common in women, whereas physical stressors have been more often reported among men. Moreover, the proportion of cases of depression in women is twice that in men, which is an important risk factor for STEMI or sudden cardiac death and increases the risk by at least 50%. Adams et al. found that the incidence of anxiety, depression, or a history of mental and neurological diseases was high in female SCAD patients [11].

Prior studies suggest that COVID-19 may be associated with an increased risk for various cardiovascular disorders, such as myocardial injury, arrhythmia, and ACS [12]. It remains unclear whether COVID-19 patients are predisposed to SCAD. However, there is no denying that the COVID-19 pandemic significantly impacted the prevalence and burden of major depressive disorder and anxiety disorders globally in 2020 [13]. This female patient was prone to anxiety in her work and other aspects of life. She did not have stable emotions and felt nervous and anxious before the SCAD attack. Her sudden SCAD may have been related to the intense psychological pressure caused by the COVID-19 epidemic.

The true prevalence of SCAD is unknown, largely due to underdiagnosis. Diagnosing SCAD requires a high index of suspicion for patients presenting with ACS. Although CAG is regarded as the gold standard to confirm the presence of SCAD, it may be difficult to distinguish a potential case of SCAD from other causes of coronary artery stenosis [14]. The booming use of new adjunctive methods of intracoronary imaging has led to increased rates of SCAD diagnosis. CAG and ancillary intracoronary imaging can help to maximize diagnostic accuracy [15]. In addition, the angiographer must be extremely careful not to cause catheter-induced coronary artery dissection.

Despite the growing awareness of SCAD, the optimal treatment of this disease remains controversial due to the lack of large-scale randomized controlled trials [16]. The ultimate goals of SCAD therapy are to relieve symptoms, improve the short-term and long-term prognosis, and prevent recurrence. The decision of whether to treat a patient with STEMI caused by SCAD with medical therapy or whether to proceed with revascularization can be complex. In general, conservative therapy is currently the recommended first-line therapy, while revascularization is recommended only for patients at high risk due to left main coronary artery dissection, ongoing ischaemia, severely limited flow, haemodynamic instability, or refractory arrhythmia [17]. For patients who do not undergo PCI, the addition of a second antiplatelet agent is controversial. In a European registry, most SCAD patients undergoing initial conservative management received dual antiplatelet therapy (DAPT). However, at the 1-year follow-up, DAPT, compared with single antiplatelet therapy, was independently associated with a higher rate of adverse cardiovascular events [18].

Long-term management mainly includes screening for FMD, monitoring for recurrence, and cardiac rehabilitation. SCAD is most prevalently associated with FMD, with up to 86% of routinely screened patients reporting characteristics of FMD [19]. Moreover, migraine headaches, anxiety, depression, and posttraumatic stress disorder have a high prevalence among post-SCAD patients, which considerably affects their quality of life [20]. Therefore, appropriate screening, treatment, and referral for these conditions are recommended. The female patient we described continues to have a good clinical status.

This paper reports a patient with STEMI who failed thrombolysis during the outbreak of COVID-19. The patient had no traditional cardiovascular risk factors and was diagnosed with SCAD. The possible inducement was excessive mental and psychological stress during the COVID-19 epidemic. This is the first reported case of STEMI caused by SCAD during the COVID-19 epidemic. The failure of thrombolysis is due to SCAD itself, which has some clinical implications. For female patients without traditional cardiovascular risk factors, especially young and middle-aged women who experience perinatal, physiological, or mental stress, when STEMI is the main clinical manifestation, we should consider the possibility of nonatherosclerotic STEMI, such as that caused by SCAD. Meanwhile, we should positively carry out CAG and further intracoronary imaging examinations to determine the aetiology and adopt corresponding treatment methods to improve the treatment success rate.

## Data Availability

The datasets used and/or analyzed during the current study are available from. the corresponding author on reasonable request.

## Abbreviations

SCAD: Spontaneous coronary artery dissection
ACS: Acute coronary syndrome
FMD: Fibromuscular dysplasia
COVID-19: Coronavirus disease 2019
STEMI: ST-segment elevation myocardial infarction
ECG: Electrocardiogram
CAG: Coronary angiography
LAD: Left anterior descending
TIMI: Thrombolysis in myocardial infarction
IVUS: Intravenous ultrasound
LVEF: Left ventricular ejection fraction
DAPT: Dual antiplatelet therapy.
