# Correction: Discussion on the causes of thrombolysis failure in a patient with STEMI: a case report

**DOI:** 10.1186/s12872-022-02950-w

**Published:** 2022-11-30

**Authors:** Lingzhi Qiu, Jia Li, Hua Yan, Hui Guo, Dan Song, Xi Su

**Affiliations:** 1 Medical College of Wuhan University of Science and Technology, Wuhan, China; 2 Department of Cardiology, Wuhan Asia Heart Hospital, Wuhan, China

**Correction: BMC Cardiovasc Disord 22, 473 (2022)**

**https://doi.org/10.1186/s12872-022-02922-0**

Following the publication of the original article [1], Lingzhi Qiu and Jia Li has been equally contributed, so the author byline and the equally contributed statement will read as follows:

Lingzhi Qiu^1,2^^†^, Jia Li^2^^†^, Hua Yan^2*^ , Hui Guo^2^, Dan Song^2^ and Xi Su^2^

^†^ Lingzhi Qiu, Jia Li has contributed equally.

The original article has been corrected.

## References

[1] Qiu L (2022). Discussion on the causes of thrombolysis failure in a patient with STEMI: a case report. BMC Cardiovasc Disord..

